# PPREMO: a prospective cohort study of preterm infant brain structure and function to predict neurodevelopmental outcome

**DOI:** 10.1186/s12887-015-0439-z

**Published:** 2015-09-16

**Authors:** Joanne M. George, Roslyn N. Boyd, Paul B. Colditz, Stephen E. Rose, Kerstin Pannek, Jurgen Fripp, Barbara E. Lingwood, Melissa M. Lai, Annice HT Kong, Robert S. Ware, Alan Coulthard, Christine M. Finn, Sasaka E. Bandaranayake

**Affiliations:** 1 Queensland Cerebral Palsy and Rehabilitation Research Centre, School of Medicine, Faculty of Medicine and Biomedical Sciences, The University of Queensland, Brisbane, Australia; 2 University of Queensland Centre for Clinical Research, Faculty of Medicine and Biomedical Sciences, The University of Queensland, Royal Brisbane and Women’s Hospital, Brisbane, Australia; 3 Digital Productivity Flagship, The Australian e-Health Research Centre, CSIRO, Brisbane, Australia; 4 School of Population Health, The University of Queensland, Brisbane, Australia; 5 Queensland Children’s Medical Research Institute, Children’s Health Queensland Hospitals and Health Service, Brisbane, Australia; 6 Royal Brisbane and Women’s Hospital, Brisbane, Australia; 7 Academic Discipline of Medical Imaging, School of Medicine, Faculty of Medicine and Biomedical Sciences, The University of Queensland, Brisbane, Australia; 8 Queensland Paediatric Rehabilitation Service, Lady Cilento Children’s Hospital, Brisbane, Australia

**Keywords:** Preterm, Magnetic resonance imaging, Neurological, Neuromotor, Neurobehaviour, Neurodevelopment, Prediction, Outcomes

## Abstract

**Background:**

More than 50 percent of all infants born very preterm will experience significant motor and cognitive impairment. Provision of early intervention is dependent upon accurate, early identification of infants at risk of adverse outcomes. Magnetic resonance imaging at term equivalent age combined with General Movements assessment at 12 weeks corrected age is currently the most accurate method for early prediction of cerebral palsy at 12 months corrected age. To date no studies have compared the use of earlier magnetic resonance imaging combined with neuromotor and neurobehavioural assessments (at 30 weeks postmenstrual age) to predict later motor and neurodevelopmental outcomes including cerebral palsy (at 12–24 months corrected age). This study aims to investigate i) the relationship between earlier brain imaging and neuromotor/neurobehavioural assessments at 30 and 40 weeks postmenstrual age, and ii) their ability to predict motor and neurodevelopmental outcomes at 3 and 12 months corrected age.

**Methods/design:**

This prospective cohort study will recruit 80 preterm infants born ≤30 week’s gestation and a reference group of 20 healthy term born infants from the Royal Brisbane & Women’s Hospital in Brisbane, Australia. Infants will undergo brain magnetic resonance imaging at approximately 30 and 40 weeks postmenstrual age to develop our understanding of very early brain structure at 30 weeks and maturation that occurs between 30 and 40 weeks postmenstrual age. A combination of neurological (Hammersmith Neonatal Neurologic Examination), neuromotor (General Movements, Test of Infant Motor Performance), neurobehavioural (NICU Network Neurobehavioural Scale, Premie-Neuro) and visual assessments will be performed at 30 and 40 weeks postmenstrual age to improve our understanding of the relationship between brain structure and function. These data will be compared to motor assessments at 12 weeks corrected age and motor and neurodevelopmental outcomes at 12 months corrected age (neurological assessment by paediatrician, Bayley scales of Infant and Toddler Development, Alberta Infant Motor Scale, Neurosensory Motor Developmental Assessment) to differentiate atypical development (including cerebral palsy and/or motor delay).

**Discussion:**

Earlier identification of those very preterm infants at risk of adverse neurodevelopmental and motor outcomes provides an additional period for intervention to optimise outcomes.

**Trial registration:**

Australian New Zealand Clinical Trials Registry ACTRN12613000280707. Registered 8 March 2013.

**Electronic supplementary material:**

The online version of this article (doi:10.1186/s12887-015-0439-z) contains supplementary material, which is available to authorized users.

## Background

Infants born very preterm (<32 weeks gestational age; GA) are at a high risk of experiencing significant motor difficulties with 10–15 % developing cerebral palsy (CP) [1], a further 40–50 % having minor motor and behavioural difficulties [2, 3] and 30–60 % experiencing cognitive difficulties at school age [4]. At least 25 % of infants follow a trajectory of typical development with no evident sequelae of their difficult neonatal course [5]. Interventions are becoming available which aim to improve outcomes for infants born very preterm, necessitating the development of tools which can firstly identify those infants at risk of adverse outcomes as early as possible, and secondly provide accurate quantitative measurement of changes that are the result of an intervention. Currently, brain Magnetic Resonance Imaging (MRI) at term equivalent age (TEA) combined with the General Movements assessment (GMs) at 3 months corrected age (CA), show the greatest predictive accuracy of motor and neurodevelopmental outcomes and CP at 1, 2 and 5 years CA [6–10].

In preterm infants imaged at TEA, structural MRI (T1 and T2 weighted images) analysed qualitatively for evidence of white and grey matter abnormalities predict motor and cognitive outcome [8, 11], motor distribution of CP [12, 13], severity of motor involvement in CP [14] and neurobehavioural development [15]. White matter injury has been identified as the predominant injury in the preterm infant brain, with lesions such as periventricular leukomalacia (PVL) and intra-ventricular haemorrhage (IVH) well described and linked to poorer outcomes and CP [8, 16]. More recently, recognition of the intercurrent and subsequent developmental disturbances in both white and grey matter as a result of the primary lesion, support the description of preterm brain injury as an ‘encephalopathy of prematurity’ [17]. Qualitative classification of grey and white matter macrostructure from structural MRI has improved prediction of outcomes, but the need for quantitative microstructural information has lead to investigation of diffusion MRI in this population [18, 19].

Diffusion MRI measures the random motion of water molecules, which is hindered and restricted by the presence of cell membranes, the cytoskeleton, and macromolecules in the brain [20]. A number of quantitative metrics can be obtained from diffusion MRI to characterise the tissue, including fractional anisotropy (FA), mean diffusivity (MD), axial diffusivity (AD), and radial diffusivity (RD) derived using the diffusion tensor model (i.e. Diffusion Tensor Imaging, DTI) [21]. These measures of the degree of restriction of diffusion (FA) and speed of diffusion (MD) change during brain development due to increasing fibre organisation, membrane proliferation, and myelination [22]. Diffusion MRI also provides estimates for the direction of the underlying white matter tracts, and, using tractography, enables the delineation of those pathways as they course through the brain.

White matter damage of prematurity is associated with increased values of MD and decreased values of FA [22, 23]. A significant correlation exists between values of FA in the corticospinal tracts and postmenstrual age (PMA) [24] and between MD and later motor impairment [25]. Higher MD values at term are associated with poorer neurodevelopmental outcomes at 2 years in preterm infants [26]. Diffusion MRI has been reported to be an independent predictor of psychomotor delay [25] and to predict CP with a sensitivity of 80 % (95 % Confidence Interval [CI] 28–100) and a specificity of 66 % (95 % CI 53–78) [25]. Associations between FA values and cognitive outcomes have been reported [27]. The use of MRI tractography to predict neurodevelopmental outcomes is not yet well established [28].

Potential limitations of diffusion imaging such as complex crossing fibre microstructure, reliability and reproducibility, are being addressed through novel diffusion MRI acquisition and analysis techniques [29]. Customized for preterm babies, they include novel pre-processing, the use of 60-direction High Angular Resolution Diffusion Imaging (HARDI), high b values and fibre orientation distribution analysis [30]. These deal with the identified need for greater accuracy of tractography and improved quantitative markers [31].

Imaging technology advances are now able to be coupled with earlier imaging, with the advent of MRI compatible incubators. Safety and feasibility have been established for MRI in the neonatal period after birth and before TEA, with the potential to provide further insights into this period of rapid brain development [32–37]. At the stage very preterm infants enter the extra-uterine environment, between the end of the second and beginning of the third trimesters, cortical neurogenesis and migration are complete, axonal and dendritic branching continue vigorously, and synaptogenesis is commencing [38, 39]. From this stage until TEA is reached, white matter increases by 5 times the original volume, cortical grey matter volume increases 4 times and cortical folding both commences and is essentially completed [15, 40]. Brain development is rapid, vulnerable to injury but also adaptive to environmental inputs that guide and consolidate developing brain connections in a process termed neuroplasticity [41].

An area of specific interest in early imaging is the cortical subplate [42]. This structure consists of neurons formed in deep grey matter neurogenic sites such as the thalamus, and arrive to lie below the cortical neurons that migrated earlier from the subventricular zone [43]. At 30 weeks gestation, the subplate reaches its peak thickness, many times thicker than the cortex, and by term has almost completely regressed [44]. This major wave of growth and death establishes the long range projections between the deep grey matter and the cortex, and the short- and long- range cortico-cortical connections that are fundamental to integration of motor and cognitive functions [45]. This information on brain structure and structural connectivity from earlier neuroimaging increases the potential of understanding the trajectory of structural brain development.

Electroencephalography (EEG) is a useful method of measuring cortical function for diagnosis and predicting later outcomes. Relationships between EEG and structural and functional connectivity have been shown throughout development in both adults and infants [46–50]. Electroencephalography signals represent cortical electrical activity measured on the scalp and can be collected non-invasively with relative ease and low cost. Electroencephalography has strong predictive capacity for outcome in the term infant with hypoxic ischaemic encephalopathy [51]. Increasing use in the preterm population, particularly in configurations using a limited number of electrodes, are evidenced with the first reports of its utility in predicting outcome [52, 53]. Multi-channel EEG, typically 10–20 channels in the newborn, is well established in clinical practice and provides information about normal and abnormal functionality of the developing brain [54]. Deeper insights are possible with further analysis of multichannel EEG [55–58]. The power and the frequency of oscillations in the cortex can be assessed using power spectral density analysis [59].

Electroencephalography is able to define the electrical activity of the neonatal brain structural network that is visualised in diffusion imaging [30, 60, 61]. The electrical activity of these networks is characterised by two alternating modes observed in the amplitudes of EEG signals: a mode associated with the self-organising, locally generated spontaneous electrical activity transients (SATs) and a mode representing the low-amplitude intervals between SATs [62, 63]. This bimodality gradually attenuates from mid gestation and activity becomes continous by term [63].

In parallel to neuroimaging and neurophysiological modalities, several clinical assessments of neuromotor, neurobehaviour and neurological function are proposed for use in the preterm period and early infancy [64]. Two systematic reviews on the clinimetric properties of such measures found Prechtl’s General Movements Assessment to have the greatest predictive accuracy of an outcome of CP [64, 65]. This neuromotor assessment evaluates spontaneous infant movement from preterm birth until 5 months CA [66]. A systematic review examining the accuracy of tests to predict CP included a meta-analysis of GMs and reported a pooled sensitivity and specificity of 98 % (95 % CI 74–100 %) and 91 % (95 % CI 83–93 %) respectively [10]. It is important to note that GMs at 3 months CA also predict severity of CP [67], cognition [68], minor neurological dysfunction [69] and behavioral and psychiatric outcome [70].

Neurobehaviour refers to an infant’s ability to self-regulate, orientate, be responsive to stimuli and sustain attention [71]. Neurobehavioural assessment in the preterm period reveals changes between birth and TEA, and differences between preterm and term infants assessed at TEA [72, 73]. Poorer neurobehavioural performance at TEA is associated with white matter abmormality on structural MRI, a range of adverse perinatal variables and predicts neurodevelopmental outcomes and CP at 18 months CA [72, 74, 75]. Components of the NICU Neonatal Neurobehavioural Scale, namely a low handling score, low movement score and high lethargy score are significantly related to an outcome of CP [75].

Neurological examination of infants offers reasonable prediction of outcomes, with sensitivity and specificity increasing as the infant progresses from the preterm period, through TEA and into the first year of life [10, 76]. Prediction of CP and motor outcome in the preterm period is relatively poor due to the presence of early transient abnormal signs with later good outcomes causing false positives and the converse resulting in false negatives [10, 77]. When neurological examination is performed before term age in preterm infants, the sensitivity for an outcome of CP is 57–86 % and specificity 45–83 % [78, 79]. At term, neurological assessment has a sensitivity of 88 % and specificity of 46 % to predict structural MRI abnormalities [80] and 68–79 % and 63–70 % to predict CP [78, 81]. In the post term period sensitivity and specificity range from 68–96 % and 52–97 % respectively [78, 81].

Perinatal factors, including growth and nutrition, have been identified as risk factors of adverse outcomes. Poor growth during the first weeks after preterm birth is a significant predictor of poor neurodevelopmental outcome [82–84]. Increased nutrient intake leads to better growth [85–87], and presumably better brain development, although this relationship is not proven. There is a need for clear evidence of the relationship between early nutrient intake and brain development in preterm infants, so that improved nutrient regimens can be designed.

Individual modalities of MRI, EEG, clinical measures, perinatal risk factors and nutrition have been evaluated in relation to later outcomes for preterm infants as described above. Combinations of modalities have been evaluated and often demonstrate improved prediction of outcomes over individual modalities alone [7, 6, 10, 88, 89]. The relationships between modalities at TEA are emerging, but to our knowledge, few studies to date have examined the relationships between early clinical measures, perinatal risk factors and nutrition, and very early imaging at 30 weeks PMA [7, 15, 72, 73, 80, 90–92]. This study aims to contribute to the understanding of brain structure-function relationships in the very early phase of the developmental trajectory, improving the ability to identify infants at risk of adverse outcomes, facilitating innovation of interventions and developing quantitative biomarkers of brain development.

### Broad aim

This prospective cohort study of infants born ≤30 weeks will investigate the relationship between brain structure (structural and diffusion MRI), brain function (neurological, neuromotor, neurobehaviour, vision and EEG), perinatal risk factors and nutrition of very preterm infants in the preterm period (30–32 weeks) and at TEA; then examine the ability of these early measures to predict motor and neurodevelopmental outcomes at 3 and 12 months CA.

### Primary aims

In a prospective cohort study of infants born at ≤30 weeks, and a term reference group, this study aims:

1. To examine the relationship between brain structure on structural and diffusion MRI, brain function on clinical measures of neurological, neuromotor and neurobehavioural performance, and perinatal risk factors at 30 and 40 weeks PMA.
2. To determine whether brain structure and function at 30 weeks PMA predicts outcomes of brain structure and function at 40 weeks PMA, 3 months CA and 12 months CA.
3. To evaluate the ability of structural and diffusion MRI and functional measures at 30 and 40 weeks PMA age to predict motor outcome at 3 months CA and motor, neurodevelopmental outcome and CP at 12 months CA.
4. To evaluate the ability of perinatal variables and social risk (socio-economic status; SES) to predict severity of motor outcome and CP at 12 months CA.

### Secondary aims

1. To examine the development of motor, sensory, visual and auditory connectivity between 30 week and 40 week MRIs in infants born preterm with and without brain lesions.
2. To examine the correlation between brain function on dense array EEG, and motor and visual outcomes at 40 weeks PMA.
3. To evaluate the ability of dense array EEG at 40 weeks PMA to predict visual outcome at 3 months CA and cognitive outcome at 12 months CA.
4. To examine the correlation between data fusion of brain functions on dense array EEG and brain structure on diffusion MRI, and motor and visual outcomes at 40 weeks PMA.
5. To evaluate the ability of data fusion of brain functions on dense array EEG and brain structure on diffusion MRI, to predict visual outcome at 3 months CA and cognitive outcome at 12 months CA.
6. To examine the relationship between preterm macronutrient intake from birth to 34 weeks and brain development at 40 weeks PMA, and determine if nutritional intake is more predictive of brain development than other maternal and neonatal risk factors.

### Hypotheses

The specific hypotheses to be tested include the following. In infants born very preterm:

1. A strong correlation exists between MRI, clinical measures and perinatal variables at 30 weeks PMA.
2. Brain structure and function at 30 weeks PMA predicts outcomes at 40 weeks PMA, 3 months CA and 12 months CA.
3. Brain structure and function at 40 weeks PMA predicts neurodevelopmental outcome at 3 and 12 months CA.
4. A strong correlation exists between EEG, clinical measures and perinatal variables at 40 weeks PMA, and 3 months and 12 months CA.

## Methods and analyses

### Design

A prospective observational cohort study of infants born very preterm with a comparison group of infants born at term.

### Ethical considerations

Ethical permission to conduct the study has been obtained from the Human Research Ethics Committees at The Royal Brisbane & Women’s Hospital (HREC/12/QRBW/245), and The University of Queensland (2012001060). The trial has been registered with the Australian New Zealand Clinical Trials Registry (ACTRN12613000280707). Participation in the study is voluntary, written informed consent for participation in the study is obtained from a parent or guardian, and families may withdraw from the study at any time without explanation.

## Study sample and recruitment

### Preterm sample

This study aims to recruit 80 preterm infants from the Neonatal Intensive Care Unit (NICU) at the Royal Brisbane and Women’s Hospital (RBWH). A research nurse will screen infant admissions for eligibility, and determine the appropriate stage to approach the family based on medical stability and approval from the treating neonatologist. Eligible families will be approached and if they express an interest in the study, they will be provided with detailed information and an explanation of the study. Parents will be given the opportunity to ask questions and discuss involvement with their treating clinician prior to making their decision. Informed written consent will be obtained from parents or guardians interested in participating and their infant will be formally enrolled.

### Inclusion criteria

Infants born at ≤30 week’s gestation, who live within 200 km of the hospital to allow for follow up hospital appointments and home visits, and have English speaking families as there is insufficient funding for translators, are eligible for this study.

### Exclusion criteria

Infants diagnosed with any congenital or chromosomal abnormality that could adversely impact neurodevelopmental outcome, and/or any contraindications to MRI, are ineligible for this study.

### Term reference sample

Twenty term born babies will be recruited from either the postnatal ward of the RBWH, or as interested volunteers by word of mouth.

### Eligibility criteria

Infants are eligible to participate in the reference sample if they are born between 38 and 41 weeks gestation following an uncomplicated pregnancy and delivery, have a birth weight above the 10th percentile, and are not admitted to neonatal intensive or special care units following their birth.

### Sample size

There are no data currently available to assess the relationship between MRI and clinical measures at 30 weeks PMA to predict motor outcome at 3 months CA and motor/neurodevelopmental outcome or CP at 12 months CA. Sample size calculations are based on a study investigating the ability of MRI at TEA, and the GMs assessment, to predict motor outcomes and CP at 12 months CA [6]. In a prospective cohort of infants born <30 weeks GA and in a total sample size of *n* = 86, MRI was classified as normal (*n* = 22), or with mild (*n* = 54), moderate (*n* = 6) or severe (*n* = 4) white matter abnormality (WMA) [93]. Infants with normal or mild WMA were grouped (*n* = 76), and infants with moderate and severe WMA were grouped (*n* = 10) [6]. We assume the same ratio (7.6 MRI normal or with mild/moderate WMA: 1 MRI with moderate/severe WMA) will be observed in this study. Of the *n* = 10 infants in the prior study that had moderate/severe WMA, *n* = 5 (50 %) developed CP [6]. If we assume that 5 % of infants with MRI normal or with mild/moderate WMA develop CP, then the study requires 69 infants to be recruited (8 with MRI with moderate/severe WMA and 61 with MRI normal or with mild/moderate WMA) in order to be able to reject the null hypothesis that the proportion of infants with CP in the two groups are equal with power = 90 %. The Type I error probability associated with this test of this null hypothesis is 0.05. In order to explore WMA earlier, at 30 weeks PMA, and its ability to predict CP at 12 months CA, an increase in the projected numbers will be required, and a further 15–20 % added to account for attrition. Consequently, the aim is to recruit a total sample size of 80 infants with full data sets.

### Perinatal data collection

An extensive record of the pregnancy, birth history, and neonatal course will be collected from the medical discharge summary. This will allow detailed description of the characteristics of the sample, allow comparison to outcomes establishing predictor variables, and to adjust for confounders.

A number of prenatal variables have been shown to impact short and long-term outcomes. Prolonged rupture of membranes, defined as spontaneous rupture of membranes ≥24 h before delivery is the most significant risk factor of a poor outcome among pregnancy history [94, 95]. Maternal antenatal corticosteroid administration reduces the risk of neonatal death and respiratory distress (complete course defined as more than 1 dose of steroids given, and 1st dose at more than 24 h and less than 8 days before birth) [94–96]. Evidence also exists for antenatal steroids protecting against cerebral haemorrhage [97]. The neuroprotective effect of magnesium sulphate administration reduces the risk of an outcome of CP (relative risk 0.68, 95 % confidence interval 0.54 to 0.87) [98]. Assisted conception is associated with adverse neurodevelopmental outcomes independent of prematurity, multiple pregnancy and gender for infants born between 22–26 weeks gestation [99]. Multiple birth status will be examined as the widely held view that singletons experience better outcomes than multiples has recently been challenged. In a population based study of *n* = 1473 born <29 weeks gestation, infants from multiple gestation pregnancies demonstrated comparable neurodevelopmental outcomes to singletons [100].

Birth history variables collected will include GA at birth, gender and birthweight. The risk of CP and adverse neurodevelopmental outcomes increases with decreasing GA at birth [101] and multiple studies report poorer outcomes for male infants [94, 102–104]. Intra uterine growth retardation (IUGR) can result in decreased cortical volume, poorer outcomes and increased risk of neonatal complications [105, 106] and babies that are small for gestational age (SGA) are at a higher risk of death, adverse neonatal outcomes and neurodevelopmental impairment [107]. Growth restriction in this study will be defined as a birth weight <10th percentile based on the Olsen growth curves.

Information will be gathered over each infant’s neonatal course from birth until discharge from hospital. Cranial ultrasound findings, specifically findings of PVL and IVH graded according to the criteria of Papile et al., 1978 will be documented, with higher grades predictive of adverse outcomes and CP [108]. Necrotising enterocolitis (NEC) is associated with poorer growth, cognitive and motor outcomes and is considered proven if the infant warranted treatment which included nil by mouth and antibiotics [95, 109]. Late onset sepsis is a significant risk factor, diagnosed by isolation of an organism from at least one blood culture and a decision to give antibiotics with therapeutic intent, from 48 hrs after birth [94, 95]. Culture proven sepsis is independently associated with an outcome of CP [110]. Postnatal corticosteroid use demonstrates an independent effect on poor outcome, in particular with behavioural outcomes and CP [94, 111, 112]. Bronchopulmonary dysplasia or chronic neonatal lung disease are independent risk factors for adverse neurodevelopmental outcomes due to recurrent episodes of hypoxia [111, 113–117]. Chronic neonatal lung disease is defined as babies born <32 weeks GA requiring any respiratory support or supplemental oxygen for a chronic pulmonary disorder at 36 weeks PMA [95]. Postmenstrual age at NICU discharge will be documented, as poorer behavioural outcomes are associated with longer length of hospital stay [118].

For each infant from birth until 34 weeks PMA, the daily intake of all nutrient-containing solutions will be recorded. Intake of protein, lipid, carbohydrate and energy for each day will be calculated by multiplying intake volumes for each solution administered by the nutrient concentration obtained from manufacturers specifications or, for breast milk, published data [119].

Socio-demographic information such as maternal and paternal education and occupation will be collected using a baseline parent questionnaire (see Additional file 1). Social and environmental factors may impact infant development, and low socio-economic status and parenting factors have been shown to adversely influence outcomes [120]. Social risk will be assessed using a score measuring six aspects of social status including: family structure, education of primary caregiver, occupation of primary income earner, employment status of primary income earner, language spoken at home and maternal age [116, 121, 122]. Each item will be scored between 0 and 2 for a total score of 12, with scores of 2 and above being considered high social risk in line with other research in this population [121, 122]. Higher social risk has been strongly associated with later behaviour problems, and independently predicts a lack of early intervention services [122, 123]. A recent systematic review found evidence that lower socio-economic status results in an additional risk of CP, over and above the risks conferred by prematurity or lower birthweight [124].

### Procedures

**Fig. 1:**
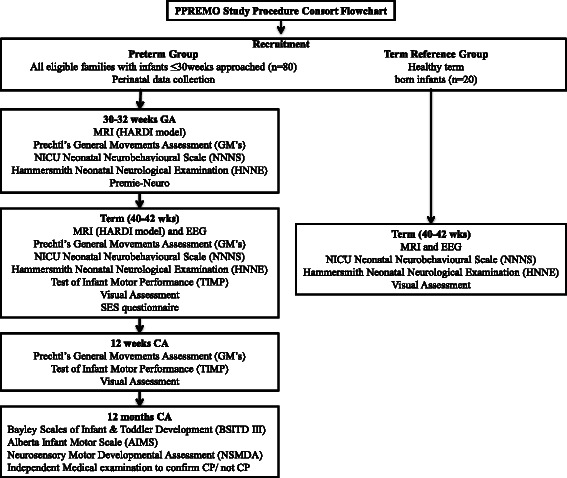
Consort Flowchart of PPREMO Study Procedure

Study procedures are depicted in Fig. 1. Participants will be recruited, consented and enrolled as described above. Between 30–32 weeks PMA, when medically stable, infants will undergo an MRI. In the event an MRI cannot be undertaken due to medical instability, MRI’s will be conducted when the infant becomes medically stable and up to a maximum age of 36 weeks PMA. This will ensure that less fragile infants are not over-represented in the sample. The following day, infants will undergo clinical assessment by an assessor blinded to GA at birth, CUS and MRI findings and any unrelated medical information, and a video recording of their spontaneous movements will be captured. As there are no established gold standard neurological or neurobehavioural assessments for use at this time point, a combination of the NICU Neonatal Neurobehavioural Scale (NNNS), Hammersmith Neonatal Neurological Examination (HNNE), and the Premie-Neuro will be used [125]. These assessments will be combined to minimise handling and modified to remove items unsuitable for administration at this age. The assessment time will be 10–15 min, conducted before a scheduled feed and cares to ensure optimum comfort and alertness. Infant cues, physiological signs of stress or distress, oxygen saturations and heart rate will be monitored throughout, and the assessment paused or discontinued where necessary. The assessment will be video recorded for independent scoring and testing of inter- and intra-rater reliability.

At TEA the family will be invited to return for their infant to undergo a second MRI and an EEG. The following day an assessor blinded to GA at birth and CUS and MRI findings will visit the family at home to undertake the clinical assessments. A video of the infant’s spontaneous movement will be recorded for later scoring of the GMs assessment, a brief assessment of visual function will be undertaken and 3 motor and neurobehavioural assessments will be administered, combined to remove duplicate items. The NNNS assessment, which is highly structured, will be completed first, followed by the few additional items of the HNNE and the Test of Infant Motor Performance (TIMP). Total assessment time will be approximately 1 h, however, the assessment will be conducted at the infant’s pace, and breaks for feeds or sleep will be undertaken as appropriate.

At 3 months CA, during a home visit, a GMs video of the infant’s spontaneous movement will be taken, and a visual assessment and the TIMP will be completed. The total assessment time will be approximately 40 min.

At 12 months CA, families will be invited back to the RBWH for follow up assessment of their child’s motor and neurodevelopmental outcome. In a telephone call prior to the appointment a research nurse will gather up to date information on the child’s current medical team, medical history since discharge, any diagnoses made and details of any interventions they have received. A paediatrician blinded to medical history will assess for signs of neurological abnormality and the presence of features of CP. A physiotherapist blinded to background history will conduct neurodevelopmental and motor assessments. As no single measure has been shown to provide conclusive data on attainment and quality of motor skills in this population, a combination of the Bayley Scales of Infant and Toddler Development III (Bayley III), the Neurosensory Motor Developmental Assessment (NSMDA) and the Alberta Infant Motor Scale (AIMS) will be performed [126]. The total assessment time will be 1–1.5 h.

## Measures

### MRI methods

#### Image acquisition

Brain MRI will be performed using a 3T (Siemens Tim Trio, Erlangen, Germany) and an MR compatible incubator with dedicated neonatal head coil (LMT Lammers Medical Technology, Lubeck, Germany). Noise from the MRI will be attenuated using Natus Mini Muffs (Natus Medical Inc., San Carlos, CA). The preterm group will have an MRI at 30–32 and again at 40–42 weeks PMA. The term group will have an MRI at 40–42 weeks PMA. All infants will be monitored with pulse oximetry and electrocardiographic monitoring. Infants will be fed, fitted with ear protection to minimize noise exposure, carefully wrapped and placed in the incubator in the scanner without sedation or anaesthesia. The total scanning duration will be approximately 45–60 min for each baby. Where possible, images impacted by significant motion artefacts will be rescanned. The MR protocol will include T1, T2 TSE, T1w MPRage, T2w HASTE and 3 echo T2 map, Arterial Spin Labelling (ASL), 30 direction diffusion weighted imaging (DWI), and 64 direction DWI sequences. Additional file 2 outlines the MRI protocol parameters. A neuroradiologist will review clinical sequences and classify white and grey matter injury [93, 127].

Quantitative T2 will be measured using a T2 image series acquired with echo times of 27, 122 and 189 ms and repetition time 10580 ms; 47 axial contiguous slices of 2.0 mm thickness will be acquired with a 144 × 180 mm field of view, a flip angle of 150°, and a 153 × 256 matrix (reconstructed to 204 × 256), resulting in voxel sizes of 0.70 × 0.70 × 2.0 mm^3^. T1-weighted magnetization prepared rapid-acquisition gradient echo volumes in the sagittal plane will be acquired with an echo time of 3.21 ms and repetition time 2100 ms; 96 sagittal slices of 1.3 mm thickness will be acquired with a 160 mm field of view, a flip angle of 9°, and a 128 × 128 matrix, resulting in voxel sizes of 1.25 × 1.25 × 1.3 mm^3^.

Diffusion images will be acquired using single-shot echo planar multi-direction diffusion-weighted sequence, employing dual bipolar diffusion gradient and double spin. This will include the acquisition of a 30 direction DWI protocol (b = 1000 s/mm^2^) and a 64 direction HARDI protocol (b = 2000 s/mm^2^). The images will be acquired per location, consisting of one low (b = 0 s/mm^2^) and the rest high (b = 1000 or 2000 s/mm^2^) diffusion-weighted images, in which the encoding gradients are uniformly distributed in space. Imaging parameters of the diffusion sequence will be: field of view 224 × 224 mm, matrix 128 × 128, repetition time 9500 ms, echo time 130 ms and flip angle of 90°. A field map for diffusion data is acquired using two 2D gradient recalled echo images (TE1/TE2 4.9/7.4 ms) to assist in correction for residual distortions due to susceptibility inhomogeneity’s (acquisition time 1 m). These sequences allow exploration of brain microstructure and function, specifically: (i) regional and global cortical surface and thickness, (ii) white matter organisation, (iii) structural connectivity of relevant areas and (iv) pre-myelination (T2).

Arterial spin labelling MRI provides a non-invasive technique to measure cerebral blood flow (CBF), although its feasibility and value in neonates is largely unknown. As the neonate’s brain rapidly grows, it is anticipated that an associated increase in CBF would occur to supply the nutrients and energy needed for the added brain weight [128]. Arterial spin labelling MRI will be performed using a PICORE Q2TIPS sequence with echo-planar imaging. Imaging parameters of the ASL scan will be: field of view 256 mm, matrix 64 × 64, repetition time 3427.5 ms, echo time 21 ms, inversion time of arterial spins (TI1) 700 ms, saturation stop time 1600 ms, total transit time of the spins (TI2) 1800 ms, tag thickness 100 mm, tag to proximal slice gap 25 mm, 17 axial slices, slice thickness 5 mm, time lag between slices 22.5 ms, and Bandwidth Per Pixel Phase Encoding time of 23.343 ms.

#### Image analysis

MRI data will be analysed using advanced image processing techniques as below.

- Structural Analysis
  T2 relaxation maps will be obtained from three T2-weighted images by first aligning all T2- weighted images to the T2-weighted image with the shortest echo time (TE = 27 ms) using rigid-body registration, followed by voxel-wise estimation of T2 employing a nonlinear least-squares fit. The T2w MR will be segmented using the MILXView neuroimaging platform with the UNC neonate atlas and ALBERT atlas used to provide initial priors and anatomical labelling [129–131]. Statistical analysis will use Regions-of-Interest and voxel based analysis techniques. Summary measures of T2 will be calculated within pathways delineated using tractography.
- Diffusion Analysis
  An extensive pre-processing and quality control procedure will be used to detect and correct image artefacts caused by involuntary head movement, cardiac pulsation, and image distortions [30]. Fractional anisotropy (FA) and mean diffusivity (MD) will be estimated from corrected diffusion data using a diffusion tensor model. Constrained spherical deconvolution implemented in MRtrix will be employed to estimate fibre orientation distribution (FOD) [132]. Whole-brain voxel based analysis of FA and MD will be performed using tract-based spatial statistics optimised for neonates [133]. Whole-brain voxel-based analysis of fibre orientation distributions will be conducted using Apparent Fibre Density (AFD) [31]. Probabilistic tractography will be performed using MRtrix. White matter pathways will be delineated using the multi-regions-of-interest approach. A number of pathways, including cortico-spinal tract, corpus callosum, superior longitudinal fasciculus and thalamic radiations, will be extracted. Summary measures of FA, MD, AFD and T2 within pathways will be calculated.
- Arterial Spin Labelling analysis
  An extensive pre-processing and quality control procedure will be used to detect and correct image artefacts caused by motion, random thermal and physiological noise, EPI distortion, spatial-temporal denoising, correction for temporal decay and partial voluming of the signal. The CBF maps will then be calculated in absolute units $\left(\frac{ml}{100g\kern0.5em 60s}\right)$, with the first equilibrium magnetization of arterial blood estimated using the calibration image (first acquired image), and GM and WM maps rescaled. Statistical analysis will use Regions-of-Interest and voxel based analysis techniques.

### EEG

Dense array EEG (dEEG) will be collected using either; i) a NicOne EEG amplifier (Cardinal Healthcare, USA) with a sampling rate of 256 Hz from 32 channels using an appropriately sized EEG cap (Waveguard, ANT-Neuro, Germany) with electrode positioning according to the international 10–20 standard, or ii) a 64-electrode high-density sensor net (HydroCel Geodesic Sensor Net, Electrical Geodesics Inc.). Each electrode is enclosed in a saline sponge, in a geodesic tension structure comprised of elastic threads EEG signals are transmitted from the sensor net electrodes to an amplifier (Electrical Geodesics Inc.) digitised and recorded via NetStation software (Electrical Geodesics Inc.).

For the EEG data i) directional relationships between channels, ii) frequency-specific amplitude fluctuations, and iii) time-varying behaviour through directional connectivity analysis and phase synchrony among channels will be examined. Electroencephalography power will be estimated in the frequency bands delta/theta (2–7 Hz), alpha (8–13 Hz), beta (14–32 Hz) to examine changes in the power and frequency of oscillations over the sensorimotor cortex as an index of corticospinal linkage and maturation [59].

The electric resting state network (eRSN) analysis will follow a multi-step procedure comprising i) pre-processing of EEG signals, ii) extracting band amplitude fluctuation envelopes at the frequency band of interest, and iii) evaluating their network characteristics within two modes of activity. Relationships between eRSN characteristics and outcome will be sought using approaches including pair-wise relationships such as mutual information measures, with testing using surrogate signals as well as different statistical testings at individual and group levels.

### Clinical measures

#### General Movements Assessment (GMs)

The GMs is a predictive and discriminative tool that involves observation of an infant’s spontaneous motor activity [66]. It can be used from preterm birth until 20 weeks CA and is carried out by videoing the infant in supine, in a calm alert state with no external stimulation. Scoring is completed from the recording with 3 full movement sequences required for pattern recognition (approximately 5 min) [66]. In the early preterm stage this may require up to an hour of video in order to select sequences of active movement, but at TEA and 12 weeks CA it may only take a few minutes. Movements are classified as normal or abnormal (poor repertoire, cramped synchronised or chaotic) in the writhing period from preterm up to 6 weeks post term. During the fidgety period from 9–20 weeks post term, fidgety movements are classified as present, abnormal or absent [66]. Infants in this study will have an assessment of their GMs in the preterm period (30–32 weeks PMA), one assessment at TEA, and one at 10–12 weeks CA. The GMs have been found to have the greatest predictive accuracy of motor outcome in two systematic reviews on the clinimetric properties of neuromotor and neurobehavioural assessment tools for use in preterm infants in the preterm period and first year of life [64, 65]. A systematic review examining the accuracy of tests to predict cerebral palsy included a meta-analysis of GMs. The pooled sensitivity and specificity were 98 % (95 % CI 73–100 %) and 91 % (95 % CI 83–95 %) respectively [10]. General Movements in the fidgety period display greater sensitivity and specificity than those in the writhing period [6, 7, 134] and have also shown an ability to predict functional severity of CP as classified by GMFCS [67]. Additionally, GMs predict cognition [68, 135, 136], minor neurological dysfunction and developmental coordination disorder [69, 137], as well as behavioral and psychiatric outcomes [70, 138].

#### The NICU Network Neurobehavioural Scale (NNNS)

The NNNS is a discriminative neurobehavioural assessment initially designed for use in prenatally substance exposed infants as part of the Maternal Lifestyle Study (MLS) [139]. It’s application for use in other high-risk infant populations including very preterm infants is now well established [64, 75, 125]. Neurobehavioural functioning is determined through evaluation of neurological and motor performance, orientation to auditory and visual stimuli, state regulation, self-soothing competence and stress signs. Forty-five items are administrated in a structured format comprising state-dependent ‘packages’, with a further 21 summary items scored. The stress/abstinence scale encompasses an additional 51 observed items. Summary scores are calculated to enable statistical analysis, and they include orientation, habituation, hypertonicity, hypotonicity, excitability, arousal, lethargy, nonoptimal reflexes, asymmetric reflexes, stress, self-regulation quality of movement and handling [140]. Training and certification is required to administer and score the assessment.

Normative data on the NNNS are available in 2 studies, with samples of 125 and 344 healthy term infants respectively, assessed within 48 h of birth [141, 142]. Data of preterm infants assessed using the NNNS at 1 month CA are available though it is important to note that the cohort is selected from the MLS sample and therefore includes infants with high social risk and drug-exposure [143]. Preterm infants display poorer neurobehaviour at TEA when compared to term controls on the NNNS [144, 73]. Significant disturbances were found in motor behaviour, tone, poorer self-regulation capacities, higher excitability scores [144], poorer orientation, lower tolerance of handling and more stress in preterm infants compared with term born infants [73]. These alterations in neurobehaviour correlated with cerebral abnormalities in white and grey matter on qualitative structural MRI [72]. Predictive validity of the NNNS has been established with neurobehaviour at term predicting motor and cognitive outcomes at 18 months, motor outcomes at 24 months and cognitive outcomes at 4.5 years [75, 145, 146]. Test-retest reliability has been established with preterm infants with correlations ranging from .30 to .44 across three time points tested (34, 40 and 44 weeks PMA) [147].

#### Hammersmith Neonatal Neurological Examination (HNNE)

The HNNE was developed for the assessment of term and preterm infants at risk of developmental delay [148–150]. It is a discriminative and predictive test that assesses posture and tone, reflexes, movements and neurobehavioural responses. It is criterion and norm referenced, with normative data from a sample of 224 healthy low-risk term infants assessed between 6 and 48 h after birth [149]. Raw scores are converted into a continuous score derived through optimality scoring with final scores ranging between 0–34, and scores <30.5 considered to be suboptimal [150]. Preterm infants have been found to have poorer scores on the HNNE compared with term born infants when assessed at TEA. In a sample of 157 infants born at <33 weeks GA mean optimality scores were 26.4 [151]. Discriminative validity was demonstrated in a normative study of a sample of 380 preterm infants (GA at birth 25–35 weeks) with a normal outcome and a sample of 85 infants who developed CP examined at TEA. Preterm infants with later outcome of CP had a greater number of suboptimal items scored compared to those preterm infants who had a normal outcome [152]. Concurrent validity has been demonstrated in 2 studies (*n* = 168 and *n* = 66), where poorer scores on the HNNE related to increasing severity of cerebral abnormality on structural MRI [72, 80]. A systematic review examining the predictive validity of the HNNE to predict an outcome of CP report a sensitivity range of 57–86 % and specificity range of 45–83 % when performed before term age (<37 weeks PMA) [78, 79]. This increases to a sensitivity range of 68–96 % and specificity range of 52–97 % when assessed in the post term period [78, 81]. Percentage agreement has been shown to be good between raters after training (>96 %) [153], however few reliability statistics are available. The infants in the present study will have the HNNE assessment at 30 weeks gestation, and TEA.

#### Premie-neuro

The Premie-Neuro is a neurological and neurobehavioural assessment tool developed by Ellison and Daily [154]. It consists of 3 subscales of 8 items each: neurologic, movement and responsiveness. Although limited published data are available for this relatively new tool, it was selected for this study for the following reasons: i) scoring of neurologic and movement subscales can be completed in even the sickest and most fragile of infants as they require minimal handling, ii) significant overlap with the HNNE and NNNS means the assessment can be scored with the addition of only 2 items overall, iii) scores are based on expected findings at differing gestational age [154]. Validity has been established for discriminating between preterm infants at high and low risk for neurodevelopmental delay, although interrater reliability was low and test–retest reliability was fair to moderate [155]. It will be scored from the combined assessment performed at 30 weeks PMA for infants in this study.

#### Neonatal visual assessment

The neonatal assessment of visual functions provides useful information on various aspects of early neonatal visual function, including ocular motility, fixation, following, acuity and attention at distance. The battery is easy to perform, does not require long training, and can be performed reliably from 32 weeks PMA [156]. It has been demonstrated to contribute to prediction of neurodevelopmental outcome in preterm babies [157–159]. The overall sensitivity and specificity of Neonatal Visual Assessment to predict 12 month CA visual scores were 90 % and 63 % respectively in 121 preterm infants [158]. In this study, infants will be assessed at TEA and 12 weeks CA.

#### Test of Infant Motor Performance (TIMP)

The TIMP is a discriminative and evaluative test of functional motor behaviour used to assess infants between the ages of 34 weeks PMA and 4 months CA [160, 161]. The test assesses the postural and selective control of movement needed for functional motor performance in early infancy and is norm referenced. Observational and elicited items are administered in a standardised procedure and the test takes 20–40 min to administer. At 12 weeks CA, the TIMP has been shown to predict 12 month motor performance with sensitivity 92 % and specificity 76 % [162] and preschool motor performance (mean age 4.75 years) with sensitivity 72 % and specificity 91 % [163]. In this study, the TIMP will be performed at TEA, and at 12 weeks CA by an assessor trained by the test author.

### Neurodevelopmental and motor outcome at 12 months

#### Medical assessment

A paediatrician experienced in infant development and diagnosis of CP will independently assess infants in this study at 12 months CA. The purpose of this assessment is to discriminate which infants are developing typically from those who are not, and to confirm diagnoses of CP or not CP [164]. It is acknowledged that 12 months CA is early to confirm a diagnosis of CP, especially in less severe cases. For this reason a structured neurological examination of posture, reflexes, muscle tone and movement will be conducted with participants classified as ‘normal’ (entirely normal neurological examination), ‘unspecified signs’ (e.g. hypotonia, asymmetric reflexes) or ‘abnormal’ (definite neurological abnormality, likely CP). In cases where CP can be confirmed, motor type and distribution will be recorded as per the SCPE guidelines [165], and functional severity established through classification with the Gross Motor Function Classification System (GMFCS) [166]. The assessment will be videoed and a second blinded assessor will perform this classification for reliability purposes.

#### Bayley Scales of Infant and Toddler Development III (Bayley III)

The Bayley III is a discriminative tool designed to assess cognitive, language and motor development, and social-emotional and adaptive behaviour [167]. It is currently the most widely used assessment tool for overall neurodevelopment in follow up studies of preterm infants between 1 and 3 years CA. It is a norm-referenced test with normative data for the cognitive, language and motor subscales taken from a sample of 1700 American infants and children [167]. Normative data for the adaptive behaviour scale was obtained independently in a sample of 1350 infants and children [167]. Normed scores of the Bayley III have a mean of 100 and a standard deviation of 15, where higher scores reflect better development. The Bayley III motor composite score correlates with the second edition of the Peabody Developmental Motor Skills (r =0.57) [167]. Reliability has been established with the average reliability coefficients for the composite scale scores ranging from .91 (Cognitive) to .93 (Language) [167]. In a systematic review of the predictive value of the Bayley III on development of very preterm infants, mental development index scores were strongly predictive of later cognitive functioning (14 studies with a total sample *n* = 1330 children), r = 0.61 (95 % CI: 0.57–0.64) [168]. Motor scale scores were only moderately predictive of later motor function (across 5 studies with a total sample of *n* = 555 children), r = 0.34 (95 % CI: 0.26–0.42). For this reason, a further two assessments which are primarily motor assessments, and have stronger psychometric properties will be used, the NSMDA and the AIMS [65]. The Bayley III involves interaction between the infant and the examiner in a standardised series of play tasks, and takes 45–60 min to administer at 12 months CA.

#### Neurosensory Motor Developmental Assessment (NSMDA)

The NSMDA is a discriminative and predictive, criterion-referenced test of gross and fine motor development [65, 169]. It examines gross and fine motor performance, neurological status, posture, balance and response to sensory input. The examiner observes and administers items and the test takes 10–30 min to complete. The results give a total score and a functional classification of motor development as normal, or with mild, moderate or severe problems of posture, movement and co-ordination. Assessment at 4 months predicts outcomes at 24 months with a sensitivity of 80 % and a specificity of 56 % [170]. Studies looking at the longer term predictive validity of the NSMDA, found assessment at 12 months had strong associations with motor and cognitive scores at 4 years [171], and NSMDA assessment at 8 months to have an 80 % sensitivity of motor outcomes at 11–13 years in extremely low birth weight infants with no apparent neurological deficit or CP [172]. The NSDMA will be used to classify each infant’s development as normal or as having mild, moderate, severe or profound motor dysfunction at 12 months CA.

#### Alberta Infant Motor Scale (AIMS)

The AIMS is a discriminative, norm-referenced tool that tests gross motor skills through the components of weight bearing, posture and antigravity movements [162, 173]. The test involves observation of the infant in prone, supine, sitting and standing and is able to be completed in this study purely through observation during the Bayley III and NSMDA assessments with no additional handling. Normative data are based on a population of 2200 term infants from 0–18 months in Alberta, Canada [174], and when recently compared with a contemporary sample of 650 Canadian infants, found to still be relevant. Normative data for preterm infants has also been published with a sample of 800 infants born at ≤32 weeks from the Netherlands [175]. Raw scores are obtained with centile ranks and age equivalent growth scores available for term and preterm infants. The AIMS has high inter-rater reliability (ICC = .98 to .99) [176, 177], and intra-rater reliability (ICC = .97-.99) [177]. Concurrent validity with the Bayley II at 12 months CA in a cohort of preterm infants has been established (*r* = .90) [177]. Although the AIMS was not designed as a predictive tool, it has moderate to excellent predictive validity. In a sample of 164 preterm infants assessed at 8 months CA, the AIMS predicted motor outcomes at 18 months CA with a sensitivity 86.4 % and specificity 93 % [178]. The suitability of using the AIMS as a discriminative and predictive tool at 12 months CA in preterm infants has been supported by a clinimetric review of neuromotor measures for preterm infants in the first year of life [65]. The AIMS will be used to classify each infant’s development as normal or suspicious/abnormal at 12 months CA in this study.

### Blinding

The researchers involved in MRI and EEG analysis (KP, JF, SER, MML, AHTK) will be blinded to GA at birth, CUS findings and clinical assessment findings. The researchers carrying out the clinical assessments and scoring (JMG, PBC) will be blinded to gestational age at birth, MRI and CUS findings. Outcome assessments at 12 months CA will be performed and scored by assessors blinded to infant perinatal history, MRI and early clinical assessment findings.

### Adverse events

There are no known health or safety risks related to any aspect of the described study. There are no known risks for MRI and no sedation will be used. The principal researchers RNB, PBC and SER will review any adverse event or unintended effect detected.

### Data analysis and statistical considerations

When models involve brain structure and function data from one time point (either 30–32 or 40–42 weeks), standard regression models will be constructed; when models use data from both 30–32 and 40–42 weeks, mixed-effects models that take into account within-infant correlation will be used. Models will be constructed using standard principles; first univariable analyses will be used to identify variables significant at the *p* < 0.15 level and these variables then entered into multivariable models one-by-one, in decreasing order of significance. At each step the current model will be compared to previous models using the likelihood ratio test. Linear regression will be used for continuous outcomes (e.g. diffusion MRI measures of FA and MD); logistic regression for binary outcomes (e.g. disability/no disability); and multinomial logistic regression for categorical outcomes with > 2 categories (e.g. NSMDA categories of normal/suspect/abnormal). Results will be presented as effect estimates and 95 % confidence intervals. The sensitivity and specificity of the predictive assessment model will be determined based on diagnosis of disability using standard definitions. Perinatal, clinical, demographic and social characteristics will be included as covariables when appropriate. Analyses will be supervised by RSW, a senior biostatistician at The University of Queensland.

## Discussion

To our knowledge, this protocol describes the first study examining the clinical correlates of early advanced brain imaging and clinical measures at 30 weeks PMA to predict motor and neurodevelopmental outcomes at 3 and 12 months CA.

The results of this study will i) establish the relationships between early clinical measures, EEG, perinatal variables and nutrition and early advanced neuroimaging at 30 weeks PMA, ii) establish which components of brain structure and function most accurately predict neurodevelopmental, motor outcomes and CP at 3 and 12 months CA, iii) accurately identify infants at risk of adverse outcomes at an earlier stage, introducing an additional window of opportunity for intervention, iv) contribute to understanding brain development between 30 and 40 weeks PMA, v) and develop robust quantitative biomarkers of brain maturation, which can then be used in the research of interventions in this population.

## Additional files

Additional file 1: **PPREMO questionnaire.** (DOC 87 kb)
Additional file 2: **Parameters of the proposed imaging sequences.** (XLSX 52 kb)

## Abbreviations

AIMS: Alberta Infant Motor Scale
ASL: Arterial Spin Labelling
Bayley III: Bayley Scales of Infant and Toddler Development, version III
CA: Corrected age
CBF: Cerebral blood flow
CI: Confidence interval
CP: Cerebral palsy
CUS: Cranial Ultrasound
DTI: Diffusion Tensor Imaging
DWI: Diffusion Weighted Imaging
EEG: Electroencephalography
GA: Gestational age
GMs: General Movements Assessment
HARDI: High Angular Resolution Diffusion Weighted Imaging
HNNE: Hammersmith Neonatal Neurological Examination
IVH: Intraventricular haemorrhage
MND: Minor neurological deficit
MR: Magnetic resonance
MRI: Magnetic resonance imaging
NNNS: NICU Neonatal Neurobehavioural Scale
NSMDA: Neuro Sensory Motor Development Assessment
PMA: Postmenstrual age
PVL: Periventricular leukomalacia
TEA: Term equivalent age
TIMP: Test of Infant Motor Performance
