# Supplementary material for: PPREMO: a prospective cohort study of preterm infant brain structure and function to predict neurodevelopmental outcome
Source: BMC Pediatr. 2015 Sep 16;15:123. doi: 10.1186/s12887-015-0439-z (PMC4572671; doi:10.1186/s12887-015-0439-z)
Supplement: Additional file 1: — PPREMO questionnaire. (DOC 87 kb) [file 12887_2015_439_MOESM1_ESM.doc]

**PPREMO**

**Prediction of PREterm Motor Outcomes**

**questionnaire for the**

**mother or Primary caregiver**

| **STUDY NUMBER:** | | | |  | |  | |  | |  |
| --- | --- | --- | --- | --- | --- | --- | --- | --- | --- | --- |
|  | | | | | | |  | |  |  |
|  |  |  |  | |  | |  | |  |  |
| **TODAY’S DATE:** |  |  |  | |  | |  | |  |  |
|  | **D** | **D** | **M** | | **M** | | **Y** | | **Y** |  |

**INSTRUCTIONS:**

**This questionnaire is designed for the mother, or primary caregiver, of the preterm child in our study. It contains a number of questions about you and your family. All your responses are totally confidential and only members of the research team will have access to this information.**

**Most questions involve ticking the most appropriate response, or writing some simple notes. Please feel free to write additional comments in the spaces provided. If you would like assistance with any part of the questionnaire, please ask one of the research team.**

Thank you again for taking the time to fill in this questionnaire.

# FAMILY DETAILS

PERSON COMPLETING THIS FORM

| 1 | What is your relationship to the child in our study? (please tick one) | | |
| --- | --- | --- | --- |
|  |  | Biological mother |  |
|  |  | Biological father |  |
|  |  | Step mother |  |
|  |  | Step father |  |
|  |  | Foster parent |  |
|  |  | Family relation (eg aunt, grandmother) |  |
|  |  | Other, please specify: |  |

| 2 | a) Are you the primary caregiver? | | | | Yes | | No |
| --- | --- | --- | --- | --- | --- | --- | --- |
|  | b) If no, who is the child’s primary caregiver. (please tick one) | | | | | | |
|  | |  | Biological mother |  | |  | |
|  | |  | Biological father |  | |  | |
|  | |  | Step mother |  | |  | |
|  | |  | Step father |  | |  | |
|  | |  | Foster parent |  | |  | |
|  | |  | Family relation (eg aunt, grandmother) |  | |  | |
|  | |  | Other, please specify: |  | |  | |

| 3 | Family Living Situation (please tick one) | | |  |
| --- | --- | --- | --- | --- |
|  |  | Child living with mother & father |  | |
|  |  | Child living with mother only |  | |
|  |  | Child living with father only |  | |
|  |  | Parents separated/divorced but both have custody rights |  | |
|  |  | Other, please specify: |  | |

| 4 |  | How many brothers and sisters does your child have? |  |
| --- | --- | --- | --- |

| 5 |  | How many children live at home? |  |
| --- | --- | --- | --- |

| 6 |  | How many adults (older than 21 years) live at home? |  |
| --- | --- | --- | --- |

| 7 | Language spoken at home (please circle one) | | | |
| --- | --- | --- | --- | --- |
|  |  | Only language spoken at home is English |  |  |
|  |  | English is one of the languages spoken at home |  |  |
|  |  | Little or no English spoken at home |  |  |
|  |  | Please indicate which is the other main language spoken at home (if not English)………………………………………………………………………………………… |  |  |

Child’s Mother, or Primary Caregiver

| 8 | How old were you at your last birthday? | Years |  |  |
| --- | --- | --- | --- | --- |

| 9 | What ethnic groups do you belong to or identify with? (e.g. Australian, Aboriginal, Italian, Greek) | |
| --- | --- | --- |
|  |  | 1. |
|  |  | 2. |
|  |  | 3. |

| 10 | Which of the following best describes your highest level of education? (please circle one) | | | |
| --- | --- | --- | --- | --- |
|  |  | Left school between 13-16 years, no formal qualifications |  |  |
|  |  | Completed Year 11 |  |  |
|  |  | High School Certificate (completed Yr 12) |  |  |
|  |  | Professional qualifications without a degree |  |  |
|  |  | University degree |  |  |
|  |  | Post graduate degree |  |  |

| 11 | a) Do you have other qualifications (e.g. trade, secretarial) | Yes | No |
| --- | --- | --- | --- |
|  | b) If yes, please specify ……………………………………………………………………………………………………………………………………………. | | |

| 12 | a) Are you working at the moment? | | | |
| --- | --- | --- | --- | --- |
|  |  | Yes, full time |  |  |
|  |  | Yes, part time (< 30hours) |  |  |
|  |  | No. |  |  |

b) If yes, please provide a title & description of the job.

__________________________________________________________________________________________________________________________________________________________________________________________________________________________________________________________________

| 13 | Are you currently living with a partner? | | | |
| --- | --- | --- | --- | --- |
|  |  | Yes, legally married |  |  |
|  |  | Yes, defacto relationship |  |  |
|  |  | No |  |  |

**If you answered “No”, please go to section 19.**

**Primary Caregiver’s Partner**

| 14 | How old was your partner at his/her last birthday? | Years |  |  |
| --- | --- | --- | --- | --- |

| 15 | What ethnic groups does your partner belong to or identify with? (e.g. Australian, Aboriginal Italian, Greek) | |
| --- | --- | --- |
|  |  | 1. |
|  |  | 2. |
|  |  | 3. |

| 16 | Which of the following best describes your partner’s highest level of education? (please circle one) | | | |
| --- | --- | --- | --- | --- |
|  |  | Left school between 13-16 years, no formal qualifications |  |  |
|  |  | Completed Year 11 |  |  |
|  |  | High School Certificate (completed Yr 12) |  |  |
|  |  | Professional qualifications without a degree |  |  |
|  |  | University degree |  |  |
|  |  | Post graduate degree |  |  |
|  |  | Don’t know |  |  |

| 17 | a) Does your partner have other qualifications (e.g. trade, secretarial) | Yes | No |
| --- | --- | --- | --- |
|  | b) If yes, please specify ………………………………………………………………………………………………………………………………………………………………………………………………………………………………………………………………………………………………………………………… | | |

| 18 | a) Is the partner working at the moment? | | | |
| --- | --- | --- | --- | --- |
|  |  | Yes, full time |  |  |
|  |  | Yes, part time (< 30hours) |  |  |
|  |  | No. |  |  |

b) If yes, please provide a title & description of the job

____________________________________________________________________________________________________________________________________________________________________________________________________________________________________________________________

| 19 | Any other comments? | Yes | No |
| --- | --- | --- | --- |
|  | b) If yes, please specify ………………………………………………………………………………………………………………………………………………………………………………………………………………………………………………………………………………………………………………………… | | |

### Thank you for completing this questionnaire
